# Implication of Progranulin and C1q/TNF-Related Protein-3 (CTRP3) on Inflammation and Atherosclerosis in Subjects with or without Metabolic Syndrome

**DOI:** 10.1371/journal.pone.0055744

**Published:** 2013-02-07

**Authors:** Hye Jin Yoo, Soon Young Hwang, Ho Cheol Hong, Hae Yoon Choi, Sae Jeong Yang, Dong Seop Choi, Sei Hyun Baik, Matthias Blüher, Byung-Soo Youn, Kyung Mook Choi

**Affiliations:** 1 Division of Endocrinology and Metabolism, Department of Internal Medicine, College of Medicine, Korea University, Seoul, Korea; 2 Department of Biostatistics, College of Medicine, Korea University, Seoul, Korea; 3 Department of Medicine, University of Leipzig, Leipzig, Germany; 4 AdipoGen, Inc., Yeonsu-gu, Incheon, Korea; University of Tor Vergata, Italy

## Abstract

**Objective:**

Progranulin and C1q/TNF-related protein-3 (CTRP3) were recently discovered as novel adipokines which may link obesity with altered regulation of glucose metabolism, chronic inflammation and insulin resistance.

**Research Design and Methods:**

We examined circulating progranulin and CTRP3 concentrations in 127 subjects with (n = 44) or without metabolic syndrome (n = 83). Furthermore, we evaluated the relationship of progranulin and CTRP3 levels with inflammatory markers and cardiometabolic risk factors, including high-sensitivity C-reactive protein (hsCRP), interleukin-6 (IL-6), estimated glomerular filtration rate (eGFR), and adiponectin serum concentrations, as well as carotid intima-media thickness (CIMT).

**Results:**

Circulating progranulin levels are significantly related with inflammatory markers, hsCRP (*r* = 0.30, *P* = 0.001) and IL-6 (*r* = 0.30, *P* = 0.001), whereas CTRP3 concentrations exhibit a significant association with cardiometabolic risk factors, including waist circumference (*r* = −0.21), diastolic blood pressure (*r* = −0.21), fasting glucose (*r* = −0.20), triglyceride (*r* = −0.34), total cholesterol (*r* = −0.25), eGFR (*r* = 0.39) and adiponectin (*r* = 0.26) levels. Serum progranulin concentrations were higher in patients with metabolic syndrome than those of the control group (199.55 [179.33, 215.53] vs. 185.10 [160.30, 204.90], *P* = 0.051) and the number of metabolic syndrome components had a significant positive correlation with progranulin levels (*r* = 0.227, *P* = 0.010). In multiple regression analysis, IL-6 and triglyceride levels were significant predictors of serum progranulin levels (*R* ^2^ = 0.251). Furthermore, serum progranulin level was an independent predictor for increased CIMT in subjects without metabolic syndrome after adjusting for other cardiovascular risk factors (*R* ^2^ = 0.365).

**Conclusions:**

Serum progranulin levels are significantly associated with systemic inflammatory markers and were an independent predictor for atherosclerosis in subjects without metabolic syndrome.

**Trial Registration:**

ClinicalTrials.gov NCT01668888

## Introduction

Inflammation is known as a pivotal pathogenic mechanism of obesity-related disorders such as type 2 diabetes, metabolic syndrome, and atherosclerosis. Adipose tissue functions as a major endocrine organ by adipokine-mediated modulation of a number of signaling cascades in target tissues that exhibit pro-inflammatory or anti-inflammatory activity [1]. Therefore, targeting the molecular mechanism that leads to dysregulated production of adipokines may provide a novel therapeutic strategy for the treatment of inflammation-related metabolic disorders and cardiovascular disease (CVD) [2].

Progranulin was first purified as a growth factor from conditioned tissue culture media [3] and is known to play a critical role in multiple physiologic and pathologic conditions, including cell growth, wound healing, tumorigenesis and neurodegenerative disease such as fronto-temporal dementia [4]. Recently, Tang el al. demonstrated that progranulin directly binds to tumor necrosis factor receptors (TNFR) and disturbs the TNF-α-TNFR interaction, suggesting its role as a physiological antagonist of TNF-α signaling [5], [6]. However, Matsubara et al. identified progranulin for the first time as a novel proinflammatory adipokine by differential proteome analysis of cellular models of insulin resistance [7]. They showed that progranulin expression was induced by TNF-α or dexamethasone and decreased with differentiation of adipocytes [7]. Moreover, ablation of progranulin prevented mice from high fat diet-induced insulin resistance and blocked elevation of an inflammatory cytokine, interleukin-6 (IL-6), in adipose tissue [7]. Previously, we have shown that serum progranulin concentrations are significantly higher in subjects with type 2 diabetes and positively correlated with macrophage infiltration in omental adipose tissue [8]. Taken together, progranulin may be an important modulator in a variety of inflammatory processes with specific effect on target tissues. Inflammation plays a crucial role in the pathophysiology of obesity-related disorders such as metabolic syndrome and atherosclerosis. However, to our knowledge, there have been no previous studies to examine circulating progranulin levels in subjects with metabolic syndrome and its relationship with carotid intima media thickness (CIMT), a useful surrogate marker for atherosclerosis.

C1q/TNF-related protein-3 (CTRP3) is a novel adipokine that is a structural and functional adiponectin paralog [9]. Peterson et al. demonstrated that administration of recombinant CTRP3 to *ob/ob* mice significantly lowered blood glucose levels by activation of the Akt signaling pathway and suppression of gluconeogenic enzymes in the liver [10]. Furthermore, CTRP3 exhibited potent anti-inflammatory properties by inhibiting the binding of lipopolysaccharides (LPS) to toll-like receptor 4 (TLR4) [11] and reducing TNF-α and IL-6 secretion in monocytic cells [12]. Recently, we developed an enzyme-linked immunosorbent assay (ELISA) for CTRP3 and reported that CTRP3 concentrations were significantly higher in subjects with type 2 diabetes or prediabetes than subjects in a normal glucose tolerance group [13].

In the present study, we aimed to clarify the clinical significance of progranulin and CTRP-3 in the context of metabolic syndrome and atherosclerosis. We examined their circulating concentrations in subjects with or without metabolic syndrome, especially after excluding individuals with type 2 diabetes or CVD. In addition, we evaluated the relationship of serum progranulin and CTRP3 levels with various cardiometabolic risk factors, such as insulin resistance, high sensitivity C-reactive protein (hsCRP), IL-6, estimated glomerular filtration rate (eGFR), and adiponectin levels, as well as CIMT, which is a reliable indicator of early carotid atherosclerosis.

## Subjects and Methods

### Study Design and Participants

Subjects who visited the Health Promotion Center of Korea University Guro Hospital for a routine health check-up were enrolled between October 2009 and March 2011 using predefined inclusion and exclusion criteria. Inclusion criteria were apparently healthy volunteers with age between 20 and 80 years. We exclude the participants had a history of CVD (myocardial infarction, unstable angina, stroke, or cardiovascular revascularization), type 2 diabetes, stage 2 hypertension (resting blood pressure, ≥160/100 mmHg), malignancy, or severe renal or hepatic disease. This study excluded subjects with a history of chronic inflammatory conditions that may affect the study results, and subjects that had taken medications that might affect inflammatory status within the last 6 months were also excluded. Participants were free of any lipid-lowering therapies for at least a 6-month period prior to enrollment. Finally, one hundred twenty-seven apparently healthy subjects who agreed to participate in the study were enrolled. Forty four subjects had metabolic syndrome and 83 participants were classified as a normal control group. Metabolic syndrome was defined according to the criteria established by the National Cholesterol Education Program Adult Treatment Panel III using the adjusted waist circumference for Asians [14]. All participants provided written informed consent and the Korea University Institutional Review Board, in accordance with the Declaration of Helsinki of the World Medical Association, approved the study protocol.

### Clinical and Laboratory Measurements

Body mass index (BMI) was calculated as weight/height^2^ (kg/m^2^) and waist circumference was measured at the midpoint between the lower border of the rib cage and the iliac crest. All blood samples were obtained the morning after a 12-hour overnight fast, and were immediately stored at −80°C for subsequent assays. Serum triglyceride and high density lipoprotein cholesterol (HDL-C) levels were determined enzymatically using a chemistry analyzer (Hitachi 747; Hitachi Inc., Tokyo, Japan). Low density lipoprotein cholesterol (LDL-C) concentrations were estimated using the Friedewald formula [15]. The glucose oxidase method was used to measure plasma glucose levelsand an electrochemiluminescence immunoassay (Roche Diagnostics, Indianapolis, IN, USA) was used to measure insulin levels. Insulin resistance was calculated with the homeostasis model assessment of insulin resistance (HOMA-IR) [16]. Estimated glomerular filtration rate (eGFR) was calculated from the Modification of Diet in Renal Disease (MDRD) study equation: (ml/min/1.73 m^2^) = 175×(Scr)^−1.154^×(Age)^−0.203^×(0.742 if female) [17]. Serum IL-6 levels were measured by ELISA (R&D Systems, Minneapolis, MN, USA). Latex-enhanced turbidimetric immunoassay (HiSens hsCRP LTIA; HBI Co., Ltd., Anyang, Korea) was used for measurement of hsCRP. Adiponectin levels were measured by ELISA (AdipoGen, Incheon, Korea). Newly-developed ELISA was used for measurement of CTRP-3 (AdipoGen, Incheon, Korea; intra- and inter-assay CVs: 7.3±1.0% and 5.8±2.7%, respectively) and progranulin (AdipoGen, Incheon, Korea; intra- and inter-assay CVs: 5.8±0.6% and 7.0±0.3%, respectively) levels.

### Measurement of CIMT

The IMT of the common carotid artery was determined using high-resolution B-mode ultrasonography (EnVisor; Philips Medical Systems, Andover, MA, USA) with a 5–12 MHz transducer. Measurements of CIMT were made using IMT measurement software (Intimascope; Media Cross Co., Tokyo, Japan) at 3 levels of the lateral and medial walls, 1–3 cm proximal to the carotid bifurcation. The mean IMT was the average value of 99 computer-based points in the region. The CIMT level in this study was calculated as the average of the left and right mean IMT values. All measurements were recorded by one trained technician who was blinded to the subjects’ anthropometric and laboratory data.

### Statistical Analysis

Each variable was assessed for a normal distribution. Data are expressed as mean ± SD or median (inter-quartile range [25%–75%]). Differences between groups were tested using an independent two-sample *t*-test or Mann-Whitney U test for continuous variables, and the Chi-square test was used to test for differences in the distribution of categorical variables. Spearman’s correlation test was performed to determine the relationships of serum progranulin and CTRP3 levels with study variables. *P*-values for the linear trend of serum progranulin and CTRP3 levels according to the tertiles in the number of metabolic syndrome components were calculated by analysis of variance (ANOVA). Multiple linear stepwise regression analysis with progranulin and CTRP3 levels as dependent variables was performed to identify the risk factors that determine serum progranulin and CTRP3 concentrations in the study subjects. The second multiple linear stepwise regression analysis was performed to determine the risk factors for the CIMT values in subjects with or without metabolic syndrome. The significance level for entry and for stay in the model was chosen to be 0.15 (the default values in SAS statistical software package). All statistical results were based on two-sided tests. Data were analyzed using SAS 9.2 (SAS Institute, Cary, NC). We regarded a *P*-value <0.05 as statistically meaningful.

## Results

### Baseline Characteristic of the Study Subjects

The clinical and biochemical characteristics of the study subjects are presented in Table 1. The metabolic syndrome group showed a significantly higher mean BMI, waist circumference, blood pressure, triglyceride, total cholesterol, fasting glucose, HOMA-IR, hsCRP, and CIMT values compared to the control group. HDL-cholesterol and adiponectin levels in the metabolic syndrome group were significantly lower than in the control group. Importantly, circulating progranulin concentrations in the metabolic syndrome group were greater than those in the control group, and almost reached a significant level (199.55 [179.33, 215.53] vs. 185.10 [160.30, 204.90], *P* = 0.051), whereas there was no significant difference in serum CTRP3 levels.

**Table 1:** Baseline Characteristics of the Study Subjects.

	Control group	Metabolic syndrome	*P*
	(n = 83)	(n = 44)	
Sex (M:F)	61:22	32:12	0.927
Age (years)	52.5±8.0	52.6±10.4	0.983
Body mass index (kg/m^2^)	24.0±2.7	27.4±3.0	<0.001
Waist circumference (cm)	82.3±10.8	91.9±6.5	<0.001
Systolic blood pressure (mmHg)	121.8±12.2	132.5±11.7	<0.001
Diastolic blood pressure (mmHg)	80.0±9.2	88.8±10.4	<0.001
AST (IU/L)	14.9±7.2	19.0±9.5	0.014
ALT (IU/L)	19.1±9.3	24.1±16.6	0.029
Total cholesterol (mmol/L)	4.0±0.9	4.3±0.9	0.044
HDL cholesterol (mmol/L)	1.1±0.3	0.9±0.2	<0.001
Triglycerides* (mmol/L)	1.0(0.7,1.4)	1.8(1.3, 2.4)	<0.001
LDL cholesterol (mmol/L)	2.4±0.7	2.5±1.0	0.604
Glucose (mmol/L)	4.4±1.0	5.1±1.1	<0.001
HOMA-IR*	1.5(83.8, 159.1)	2.0(1.7, 3.2)	<0.001
eGFR* (mL/min/1.73 m^2^)	107.7(83.8, 159.1)	98.3(84.7, 127. 7)	0.229
IL-6* (pg/mL)	0.11(0.07, 0.13)	0.13(0.09, 0.16)	0.122
hsCRP* (mg/dL)	0.43(0.24, 0.97)	0.87(0.42, 2.63)	0.001
Adiponectin (µg/mL)	9.61±4.13	7.93±2.83	0.018
CTRP3*(ng/mL)	332.9(287.1, 402.9)	310.0(269.7, 369.9)	0.123
Progranulin*(ng/mL)	185.1(160.3, 204.9)	195.6(179.3, 215.5)	0.051
Carotid IMT (mm)	0.70±0.12	0.77±0.20	0.022

Data are expressed as mean ± standard deviation or median (inter-quartile range).

*P*-values were calculated by an independent two-sample *t*-test, Mann–Whitney U-test, or Pearson’s chi-square test.

AST, aspartate aminotransferase; ALT, alanine aminotransferase; HDL, high-density lipoprotein; LDL, low-density lipoprotein;HOMA-IR, homeostasis model assessment of insulin resistance; eGFR, estimated glomerular filtration rate;IL-6, interleukin-6; hsCRP, high-sensitivity C-reactive protein;CTRP-3, C1q/TNF-related protein-3; IMT, intima-media thickness.

* Non-normally distributed.

### Correlation of Circulating Progranulin and CTRP3 Concentrations with Cardiometabolic Risk Factors

**Figure 1:**
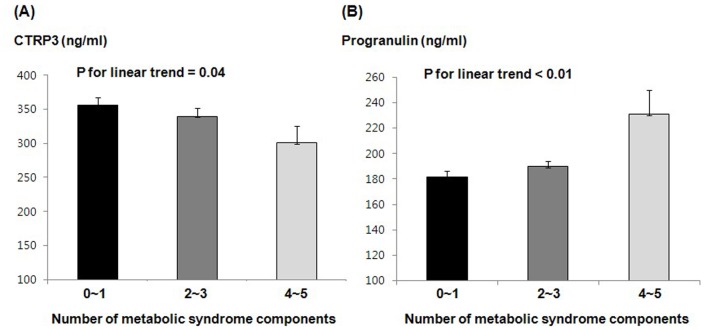
Serum C1q/TNF-related Protein-3 (CTRP3) (A) and Progranulin Levels (B) According to Number of Metabolic Syndrome Components.

Serum progranulin levels had significant positive correlations with serum hsCRP and IL-6 levels (*r* = 0.304, *P* = 0.001 and *r* = 0.300, *P* = 0.001, respectively), but had no significant correlations with various metabolic parameters, including BMI, waist circumference, glucose tolerance, blood pressure, and lipid profiles (Table 2). On the other hand, circulating CTRP3 levels were significantly negatively correlated with waist circumference, diastolic blood pressure, total cholesterol, triglyceride, and fasting glucose levels, and positively correlated with age, eGFR, and serum adiponectin levels. However, serum CTRP3 concentrations had no significant correlation with serum hsCRP or IL-6 levels. Interestingly, the number of metabolic syndrome components had a significant positive relationship with circulating progranulin levels (*r* = 0.227, *P* = 0.010) and a negative correlation with CTRP3 levels (*r* = −0.175, *P* = 0.050). Moreover, serum progranulin levels increased significantly according to the number of metabolic syndrome components (*P* for linear trend <0.01, Figure 1), whereas CTRP3 serum concentration decreased significantly (*P* for linear trend = 0.04, Figure 1). In multiple stepwise linear regression analysis, IL-6 (*P* = 0.01) and triglyceride (*P*<0.001) levels were significant determining factors for serum progranulin levels (*R* ^2^ = 0.251), whereas sex (*P*<0.001), triglyceride levels (*P*<0.001) and LDL-cholesterol levels (*P* = 0.02) were significant decisive factors for circulating CTRP3 concentrations (*R* ^2^ = 0.321) (Table S1).

**Table 2:** Spearman Correlation of Serum Progranulin and CTRP3 with Various Metabolic Parameters.

	CTRP3		Progranulin	
	*r*	*P*	*r*	*P*
Sex	0.476	<0.001	0.127	0.155
Age	0.240	0.007	0.016	0.861
Body mass index	−0.129	0.149	0.126	0.159
Waist circumference	−0.214	0.016	0.098	0.271
Systolic blood pressure	−0.076	0.397	0.121	0.175
Diastolic blood pressure	−0.207	0.020	0.144	0.107
AST	−0.126	0.159	0.096	0.283
ALT	−0.138	0.123	0.136	0.128
Total cholesterol	−0.245	0.006	0.041	0.648
HDL-cholesterol	0.093	0.302	−0.079	0.378
Triglycerides	−0.338	<0.001	0.041	0.648
LDL-cholesterol	−0.135	0.131	−0.003	0.973
Fasting glucose	−0.198	0.026	0.134	0.134
HOMA-IR	−0.108	0.321	0.166	0.123
eGFR	0.392	<0.001	−0.023	0.800
IL-6	0.125	0.162	0.300	0.001
hsCRP	−0.050	0.574	0.304	0.001
Adiponectin	0.260	0.003	−0.047	0.597

CTRP-3, C1q/TNF-related protein-3; AST, aspartate aminotransferase; ALT, alanine aminotransferase; HDL, high-density lipoprotein; LDL, low-density lipoprotein;HOMA-IR, homeostasis model assessment of insulin resistance; eGFR, estimated glomerular filtration rate;IL-6, interleukin-6; hsCRP, high-sensitivity C-reactive protein;IMT, intima-media thickness.

### Determinant Factors Associated with CIMT Values in Subjects with or without Metabolic Syndrome

Spearman correlation analysis also showed that serum progranulin level was significantly positively correlated with CIMT in subjects without metabolic syndrome (*r* = 0.236, *P* = 0.035). Similarly, the results of our multiple stepwise linear regression analysis showed that age, sex, BMI, HDL-cholesterol, and circulating progranulin (*P* = 0.039) levels were significant predictors for CIMT in subjects without metabolic syndrome (*R* ^2^ = 0.365)(Table 3). On the other hand, age, diastolic blood pressure, and LDL-cholesterol levels were significant determining factors for CIMT in the metabolic syndrome group (*R* ^2^ = 0.433).

**Table 3:** Multiple Stepwise Regression Analyses for Determinant Factors Associated With Carotid Intima-Media Thickness in Subjects With or Without Metabolic Syndrome.

	B	SE	*P*
Subjects without metabolic syndrome (*R^2^* = 0.365)			
(Constant)	0.014	0.147	0.927
Age	0.008	0.001	0.000
Sex	−0.045	0.026	0.085
Body mass index	0.010	0.004	0.019
HDL-cholesterol	−0.002	0.001	0.059
Progranulin	0.001	0.000	0.039
Subjects with metabolic syndrome (*R^2^* = 0.433)			
(Constant)	−0.268	0.268	0.324
Age	0.011	0.002	0.000
Diastolic blood pressure	0.004	0.002	0.122
LDL-cholesterol	0.001	0.001	0.142

Independent variables for mean IMT: age, sex, body mass index, systolic blood pressure, diastolic blood pressure, HDL-cholesterol, triglycerides, LDL-cholesterol, fasting glucose, hsCRP, adiponectin and progranulin levels.

SE, standard error; *R* ^2^, coefficient of determination.

## Discussion

The present study showed that circulating progranulin levels mainly have a significant relationship with inflammatory markers, such as hsCRP and IL-6, whereas circulating CTRP3 concentrations exhibit a significant association withcardiometabolic risk factors, including waist circumference, diastolic blood pressure, fasting glucose levels, lipid profiles, eGFR, and adiponectin levels. Furthermore, we found that serum progranulin level is an independent marker for carotid atherosclerosis in subjects without metabolic syndrome, even after adjusting for other cardiovascular risk factors.

Progranulin, also known as proepithelin or acrogranin, is a widely-expressed, 593 amino acid secreted glycoprotein [18]. Reported biological activities of progranulin include growth factor-like activities, modulation of immune responses, and neuronal effects [19]. Recently, Tang et al. found that progranulin is a ligand of TNFR and the anti-inflammatory effects of progranulin are mainly mediated by inhibition of TNF-α-activated intracellular signaling; Treatment of bone marrow-derived macrophages with recombinant progranulin inhibited TNF-α-induced phosphorylation of p38, c-jun N-terminal kinase (JNK), and the mitogen-activated protein kinase (MAPK) family, and impaired NF-κB nuclear translocation [5]. They have shown that progranulin prevents mice from inflammatory arthritis by blocking interaction with TNF-α [5]. However, not all the actions of progranulin on inflammatory cells are inhibitory, and the interactions between progranulin and inflammation were reported to be more complicated in some previous reports. During the inflammatory process, progranulin is digested into smaller peptides, called granulins, that are pro-inflammatory and neutralize the anti-inflammatory effect of intact progranulin [6]. Moreover, Okura et al. reported that progranulin increased the expression of TNF-α and IL-1β in human monocyte-derived macrophages [20]. In a cutaneous wound, progranulin promoted the accumulation of neutrophils and macrophages, suggesting the chemotactic activity of progranulin for inflammatory cells [21]. Furthermore, we previously reported that elevated progranulin serum concentrations were positively associated with omental adipose tissue macrophage infiltration and increased in subjects with type 2 diabetes, suggesting progranulin as a chemoattractant molecule [8]. These results support the hypothesis that progranulin may play dual roles in the inflammatory process and may exert anti-inflammatory or pro-inflammatory functions depending on the target tissue. In this study, which included subjects without diabetes, circulating progranulin levels had a significant positive correlation with serum hsCRP and IL-6 levels, reflecting chronic subclinical inflammation. Very recently, progranulin was identified as a novel adipokine that mediates high fat diet-induced insulin resistance. In that study, insulin resistance induced by progranulin was significantly improved by a neutralizing antibody against IL-6, implicating IL-6 as a mediator of progranulin-induced insulin resistance in adipocytes [7]. Interestingly, multiple regression analysis in this study showed that serum IL-6 level was an independent determining factor for circulating progranulin levels, even after adjusting for other confounding risk factors.

Our study demonstrates that serum progranulin is an independent maker for subclinical atherosclerosis, represented as CIMT. Atherosclerosis is a chronic inflammatory process resulting from the interaction of modified lipoprotein, macrophages, and the normal cellular elements of the arterial wall [22]. Growing evidence suggests that various adipokines are directly involved in the process of atherosclerosis [23]. An immunohistochemical analysis of human carotid endoatherectomy specimens indicated that intimal vascular smooth muscle cells and some macrophages in human atherosclerotic plaque express progranulin [24]. Progranulin in the plaque would be cleaved into granulins, which increase IL-8 levels and drive the migration of inflammatory cells to the vessel wall [24]. A recent clinical study reported that serum progranulin levels were significantly higher in subjects with non-alcoholic fatty liver disease (NAFLD), which is now regarded as an independent cardiovascular risk factor, and were associated with adverse lipid profiles [25]. In the present study, an independent association between CIMT and serum progranulin levels, together with age, sex, BMI, and HDL-cholesterol levels, was found in subjects without metabolic syndrome. On the other hand, in subjects with metabolic syndrome, age, diastolic blood pressure, and LDL-C levels were determining risk factors for CIMT. Although the exact explanation for this result is not clear, progranulin may have a major influence on the early stages of atherosclerosis, which may be associated with inflammation rather than the classical cardiovascular risk factors.

CTRP3 is a newly-discovered adipokine whose structure contains a 246 amino acid sequence protein, and is regarded as an adiponectin paralog [26]. Recombinant CTRP3 reduced glucose output in cultured rat hepatoma cells by suppressing gluconeogenic genes [10], significantly inhibited LPS-induced IL-6 and TNF-α secretion in THP-1 cells, and reduced NF-κB p65 activity [12]. These results suggest the biological relevance of CTRP3’s antidiabetic and anti-inflammatory properties. In the present study, we included subjects without diabetes, and circulating CTRP3 showed significant negative correlations with metabolic risk factors, including waist circumference, serum triglyceride, and glucose levels. We also observed a significant positive correlation between serum CTRP3 levels and circulating adiponectin concentrations. However, in our previous study, serum CTRP3 levels were elevated in subjects with type 2 diabetes and showed significant positive correlation with cardiometabolic risk factors such as waist-to-hip ratio, glucose, and hsCRP levels [13]. Although the reason or these discordant results could not be clarified in the present study, we could suggest several hypotheses to explain this result. First, the paradoxical increase of CTRP3 in the subjects of type 2 diabetes might be originated from a compensatory mechanism to overcome the metabolic stress or resistance. Hormone resistance to the effects of insulin, leptin, and fibroblast growth factor 21 (FGF21) has been reported in diabetes and obesity [27], [28]. In our previous study, a subgroup analysis that included only subjects without diabetes showed a similar tendency to the results of this study, although the negative relationship between CTRP3 level and cardiometabolic risk factors did not reach a significant level due to the insufficient number of subjects [13]. Secondly, the biological function of CTRP3 can be different according to glucose tolerance status. Kopp et al. showed that CTRP3 reduced the LPS induced release of macrophage migration inhibitor factor in non-diabetic controls, whereas no effects in type 2 diabetic subjects [11]. Lastly, the participants of the previous study included type 2 diabetes, so many people had been taken various kinds of medications which may affect the circulating CTRP3 levels. Further studies to clarify the underlying mechanism for the regulation of CTRP3 should be followed. Interestingly, circulating CTRP3 levels had significant negative correlations with various metabolic risk factors such as waist circumference, diastolic blood pressure, triglycerides, and fasting glucose, whereas serum progranulin levels showed significant positive relationship with inflammatory markers such as hsCRP and IL-6. These results suggest that CTRP3 may be more closely related with metabolic parameters, whereas progranulin may be more closely associated with inflammatory parameters in humans.

There are some limitations to this study. First, because it was a cross-sectional study, no causality could be defined. It is not clear whether circulating progranulin and CTRP3 levels are causative factors or markers of the pathogenesis of inflammatory diseases and atherosclerosis. Secondly, this study enrolled only Asian subjects without diabetes or CVD, so the relationship of serum progranulin and CTRP3 levels to metabolic risk factors should be further evaluated in other ethnic populations and in the context of different interventions for the treatment of diabetes and CVD. Thirdly, the subjects with renal insufficiency, defined as an eGFR <60 (mL/min/1.73 m^2^), were very few in this cohort (n = 2). Therefore, to clarify the relationship of renal dysfunction with CTRP3, further studies including the subjects with renal impairment should be followed. Lastly, the data about smoking, alcohol, and exercise were not available in this cohort, so we could not adjust the effect of these lifestyle factors.

In conclusion, this study showed that serum progranulin levels had a significant positive relationship with hsCRP and IL-6 concentrations. Furthermore, serum progranulin level was an independent determining risk factor for carotid atherosclerosis in subjects without metabolic syndrome. On the other hand, circulating CTRP3 concentration had a significant association with cardiometabolic risk factors, such as obesity, glucose levels, lipid parameters, eGFR, and adiponectin levels. Further experimental and prospectively-designed studies should be performed to clarify the influences of these two novel adipokines, progranulin and CTRP3, on the pathogenesis and outcomes of chronic metabolic disorders and cardiovascular disease.

## Supporting Information

Table S1 **Multiple Stepwise Regression Analyses for Determinant Factors Associated with Serum Progranulin and CTRP3 Levels.**
(DOC)
