# Supplementary material for: Implication of Progranulin and C1q/TNF-Related Protein-3 (CTRP3) on Inflammation and Atherosclerosis in Subjects with or without Metabolic Syndrome
Source: PLoS One. 2013 Feb 7;8(2):e55744. doi: 10.1371/journal.pone.0055744 (PMC3567086; doi:10.1371/journal.pone.0055744)
Supplement: Table S1 — Multiple Stepwise Regression Analyses for Determinant Factors Associated with Serum Progranulin and CTRP3 Levels. (DOC) [file pone.0055744.s001.doc]

|  | **B** | **SE** | ***P*** |
| --- | --- | --- | --- |
| **Dependent Variable: Progranulin** (*R^2^* = 0.251) | | | |
| (Constant) | 158.320 | 6.410 | < 0.01 |
| Triglyceride | 0.199 | 0.036 | < 0.01 |
| IL-6 | 62.094 | 23.218 | < 0.01 |
| **Dependent Variable: CTRP3** (*R^2^* = 0.321) | | | |
| (Constant) | 394.578 | 26.363 | < 0.01 |
| Gender | 97.931 | 15.979 | < 0.01 |
| Triglyceride | -0.238 | 0.074 | < 0.01 |
| LDL-cholesterol | -0.553 | 0.228 | 0.02 |

Independent Variables: age, gender, body mass index, systolic blood pressure, diastolic blood pressure, HDL-cholesterol, Triglyceride, LDL-cholesterol, fasting glucose, eGFR, hsCRP, IL-6, adiponectin

SE, standard error; *R*^2^, coefficient of determination
